# Association of Comorbidity and Inflammatory and Nutritional Markers with Epilepsy and Seizure Frequency

**DOI:** 10.3390/nu17111847

**Published:** 2025-05-28

**Authors:** Demet Aygun, Hafize Uzun

**Affiliations:** 1 Department of Neurology, Faculty of Medicine, Istanbul Atlas University, 34408 Istanbul, Turkey; 2 Department of Medical Biochemistry, Faculty of Medicine, Istanbul Atlas University, 34408 Istanbul, Turkey; huzun59@hotmail.com

**Keywords:** Charlson comorbidity index, epilepsy, NLR, PLR, SII, PNI

## Abstract

**Background:** Epilepsy is a chronic neurological disorder frequently influenced by systemic inflammation, nutritional status, and comorbid conditions, which may worsen seizure outcomes. Given the increasing recognition of these factors in disease progression, this study aimed to investigate the relationship between the Modified Charlson Comorbidity Index (mCCI), inflammatory hematological parameters, and the prognostic nutritional index (PNI) with seizure frequency and clinical prognosis in patients with epilepsy. **Methods:** A total of 159 participants were enrolled between January 2021 and January 2023, including 53 healthy controls (mean age: 44 ± 14.2 years; female: 21, male: 32), 53 epilepsy patients without comorbidity (mean age: 33 ± 12.5 years; female: 28, male: 25), and 53 epilepsy patients with comorbidities (mean age: 56.2 ± 13.8 years; female: 22, male: 31). The participants were divided into three groups: 53 patients with isolated epilepsy, 53 patients with epilepsy and comorbid conditions, and 53 healthy individuals with no known diseases, matched for age and sex with the patient groups, who presented for routine check-ups. The mCCI was calculated for patients with comorbid epilepsy. Inflammatory hematological parameters and the PNI were assessed in all participants using previously obtained complete blood count data. **Results:** Inflammatory markers such as white blood cell count, neutrophil count, C-reactive protein (CRP), platelet-to-lymphocyte ratio (PLR), neutrophil-to-lymphocyte ratio (NLR), systemic immune-inflammation index (SII), and mean platelet distribution width (PDW) were significantly higher in epilepsy patients with comorbidities compared to other groups. Epilepsy patients with comorbidities had a higher seizure frequency compared to those without comorbidities (75.5% vs. 54.7%, *p* < 0.001). The PNI was lowest in epilepsy patients with comorbidities, showing a significant difference between all groups (*p* < 0.001). High comorbidity burden increased seizure risk by 4.56 times (95% CI: 1.30–16.01), each unit increase in the SII raised the risk by 1.13 times (95% CI: 1.08–1.19), and each unit decrease in the PNI increased the risk by 1.14 times (OR = 0.88, *p* < 0.001). Cerebrovascular disease and hemiplegia were also significant risk factors, increasing seizure risk by 4.15 and 4.48 times, respectively. **Conclusions:** Our study demonstrates that inflammatory hematological parameters, particularly SII and MCCI scores, are elevated in epilepsy patients and further increase with comorbidities. These markers are strongly associated with seizure occurrence, highlighting the prognostic significance of systemic inflammation and comorbidity burden in epilepsy. Given the frequent observation of low PNI values in patients with comorbid conditions, which may reflect compromised nutritional status, and given associations suggest a role in poor clinical outcomes, comprehensive management is essential. Monitoring the PNI and SII may help stratify high-risk patients for targeted nutritional and anti-inflammatory interventions.

## 1. Introduction

Epilepsy is a chronic neurological disorder that affects individuals of all ages and remains one of the most prevalent neurological diseases worldwide, with an estimated 50 million patients, according to the World Health Organization (WHO) [1,2]. Epilepsy affects over 50 million people globally, with a higher prevalence in low- and middle-income countries (LMICs) compared to high-income countries (HICs). It is most common in children, particularly in LMIC regions, where 90% of cases are in those under 20. The incidence is highest in early childhood, influenced by factors like perinatal complications. The Global Burden of Disease Study 2021 highlights the ongoing significant health burden of epilepsy [3]. The pathophysiological mechanisms of epilepsy involve a range of molecular and cellular alterations that lead to neuronal hyperexcitability and hypersynchrony. At the molecular level, dysfunctions in ion channels, alterations in neurotransmitter systems (particularly glutamatergic and GABAergic pathways), and disrupted intracellular signaling contribute significantly to seizure development and propagation. Additionally, neuroinflammation, synaptic reorganization, and gliosis are key components in epileptogenesis. These mechanisms underscore the complexity of epilepsy and support the need for molecularly targeted therapeutic strategies. Elucidating the molecular and cellular mechanisms of epilepsy through diverse approaches—such as animal models, non-coding RNAs, and optogenetics—and integrating these insights into innovative therapeutic strategies holds promise for addressing the current unmet needs in epilepsy treatment [4].

Despite significant advancements in diagnosis and treatment, nearly 30% of epilepsy patients continue to experience seizures [5]. The pathophysiology of epilepsy involves the transformation of normal neuronal networks into hyperexcitable circuits due to disruptions in extracellular ion homeostasis, alterations in energy metabolism, receptor function, and neurotransmitter uptake [6,7,8,9,10]. Given the complexity of these mechanisms, various pathological conditions, including comorbidities, may influence seizure occurrence and overall disease prognosis.

Accumulating evidence suggests that comorbid conditions negatively impact epilepsy prognosis, with approximately half of adult epilepsy patients presenting with at least one comorbidity [11,12,13,14]. These comorbidities have been associated with poorer treatment response, prolonged hospital stays, and increased mortality rates [15,16,17]. For instance, neuropsychiatric disorders have been linked to an elevated risk of seizures and reduced survival in elderly epilepsy patients, while psychiatric comorbidities have been shown to impair treatment outcomes in pediatric cases [11,13].

The Modified Charlson Comorbidity Index (mCCI) is widely employed to quantify the effect of comorbid conditions on disease severity, functioning as a prognostic tool to estimate survival and guide clinical decision-making in individuals with multiple comorbidities [18,19]. In addition to comorbidities, emerging evidence suggests that demographic factors and inflammatory biomarkers may also influence epilepsy prognosis. Markers such as the total white blood cell count, neutrophil-to-lymphocyte ratio (NLR), eosinophil-to-lymphocyte ratio (ELR), neutrophil-to-monocyte ratio (NMR), and lymphocyte-to-monocyte ratio (LMR) have been recognized as indicators of chronic inflammation [20,21]. Moreover, the platelet-to-lymphocyte ratio (PLR) and mean platelet volume (MPV) have been identified as potential markers of systemic inflammation, particularly when evaluated alongside NLR [22].

Nutritional status is closely linked to immune function and overall health. The prognostic nutritional index (PNI), calculated using serum albumin and lymphocyte count, serves as a simple and effective marker of immuno-nutritional status [23]. Albumin, the most abundant plasma protein, reflects both nutritional condition and inflammatory activity due to its antioxidant and anti-inflammatory roles [23]. The PNI has been shown to predict clinical outcomes and identify malnutrition in various conditions, including cardiovascular diseases, such as coronary artery disease and heart failure. Low PNI values are associated with poor prognosis and reduced survival [24]. In the context of epilepsy, particularly among patients with comorbid conditions, low PNI values may indicate compromised nutritional status and heightened systemic inflammation. These factors and associations can suggest a role in seizure frequency and severity as well as poorer overall health outcomes. Therefore, monitoring the PNI in epilepsy patients could serve as a valuable indicator for identifying those at higher risk of adverse clinical outcomes, thereby guiding targeted nutritional and anti-inflammatory interventions.

Although comorbidities and systemic inflammation are increasingly recognized as contbutors to epilepsy prognosis, there remains limited data evaluating the combined impact of these factors alongside nutritional status, which is a critical but often overlooked determinant of immune function and disease outcomes in epilepsy. Prior studies have generally assessed these variables in isolation, limiting their clinical applicability in developing comprehensive prognostic models.

This study aims to fill this gap by investigating the interplay between comorbidity burden (measured by the mCCI), inflammatory hematological parameters (such as the NLR, PLR, and SII), and nutritional status assessed by the PNI in relation to seizure frequency and clinical prognosis in epilepsy patients. By placing particular emphasis on the role of nutritional health as an immunomodulatory factor, the study seeks to provide a more integrative understanding of the biological mechanisms influencing seizure control. These findings may support the use of the PNI as a practical tool for identifying at-risk individuals and promoting early nutritional and clinical interventions in the management of epilepsy.

## 2. Material and Methods

This study was conducted as a retrospective case-control study at the Department of Neurology, Istanbul Atlas University Medical Faculty Hospital. The study was designed in accordance with the Declaration of Helsinki and good clinical practice guidelines. It was approved by Istanbul Atlas University Non-Interventional Scientific Research Ethics Committee (Ethics Committee approval date: 15 February 2023; number: E-226686390-050.99-24273).

### 2.1. Study Population

A total of 159 participants were included: 53 patients with isolated epilepsy, 53 patients with epilepsy and comorbid conditions, and 53 healthy controls. All patients were selected from individuals who presented to and were followed at the Neurology Outpatient Clinic of Istanbul Atlas University Medical Faculty Hospital between January 2021 and January 2023. The control group consisted of age- and sex-matched healthy individuals who presented to the same institution for routine health check-ups during the same period and had no known chronic diseases or regular medication use. All patients had received regular epilepsy treatment for at least one year. Exclusion criteria for the patient groups included pregnancy, recent surgery within the past month, or a history of infection during the same period. For the control group, individuals with any documented chronic disease or current medication use were excluded.

### 2.2. Definition of Epilepsy

Specifically, epilepsy was defined according to the criteria of the International League Against Epilepsy (ILAE), which included the following [25]: (1) at least two unprovoked (or reflex) seizures occurring more than 24 h apart; (2) one unprovoked seizure and a probability of further seizures similar to the general recurrence risk (at least 60%) after two unprovoked seizures, occurring over the next 10 years; or (3) diagnosis of an epilepsy syndrome.

Data on participants’ age, sex, duration of epilepsy, seizure frequency, epilepsy type, electroencephalography (EEG) findings, magnetic resonance imaging (MRI) results, and antiepileptic drug use were obtained from medical records.

### 2.3. Modified Charlson Comorbidity Index (mCCI)

The mCCI includes 19 comorbid diseases, and a weight score is created for each comorbid condition by considering the annual relative mortality risk. Comorbidities are scored between 1 and 6 points in terms of mortality risk and disease severity, and the total CCI score is obtained by adding the scores. Subsequently, in the mCCI, a total score is calculated by adding one point for every 10 years of age over 40. In the scoring, “0” points are considered for no disease; “1” point for congestive heart failure, coronary artery disease, peptic ulcer disease, peripheral vascular disease, chronic pulmonary disease, diabetes mellitus (DM), cerebrovascular disease, liver disease (mild), dementia, and connective tissue disease; “2” points for DM (accompanied by three organ damage), renal disease (moderate or severe), hemiplegia, non-metastatic solid tumor, leukemia, lymphoma, and multiple myeloma; “3” points for liver disease (moderate or severe); and “6” points for metastatic solid tumor and acquired immune deficiency syndrome. The lowest possible score on the mCCI is 0, and the highest score is 37. Scoring is calculated according to the 12-month relative mortality risk of each disease [26,27]. Hypertension and hyperlipidemia, which are not included in the index, were determined and recorded for separate risk factors.

### 2.4. Hematological Parameters

Data were recorded retrospectively from patient files. It was ensured that the blood samples were taken from the antecubital vein after 8 h of fasting and were studied with the same devices in the same laboratory. NLR levels were calculated by dividing the neutrophil count by the lymphocyte count, PLR levels were calculated by dividing the platelet count by the lymphocyte count, NMR levels were calculated by dividing the neutrophil count by the monocyte count, and LMR levels were calculated by dividing the lymphocyte count by the monocyte count. Systemic immune-inflammation index (SII) was calculated by multiplying the total platelet count by the neutrophil-to-lymphocyte ratio. The PNI levels of the patients were calculated using the following formula: PNI = 10 × serum albumin level (g/dL) + 0.005 × total lymphocyte count (mm^3^) [28]. PNI values were classified as normal for those ≥35 and low for those <35.

### 2.5. Statistical Analysis

Data analysis was conducted using IBM SPSS Statistics for Windows, version 20.0 (IBM Corp., Armonk, NY, USA). Numerical variables with a normal distribution, as verified by Kolmogorov–Smirnov tests, are presented as mean ± standard deviation (SD), while non-normally distributed variables are expressed as median values with interquartile ranges (25th–75th percentile). For group comparisons, the Student’s *t*-test was used for normally distributed data, and the Mann–Whitney U test was applied for data not meeting normality assumptions. Categorical variables are shown as frequencies and percentages, with comparisons between groups performed using the Chi-square or Fisher’s exact tests. To identify independent predictors of seizure risk, a multivariable logistic regression analysis was carried out using the backward Wald method. The receiver operating characteristic (ROC) curve analysis was applied to assess diagnostic performance, and the results of area under the curve (AUC), standard error (SE), and sensitivity and specificity are reported. The optimal threshold value of the inflammation indices, PNI, and MCCI in predicting seizure risk was determined by the Youden index method. Statistical significance was set at *p* < 0.05 (*) for all tests.

## 3. Result

**Figure 1:**
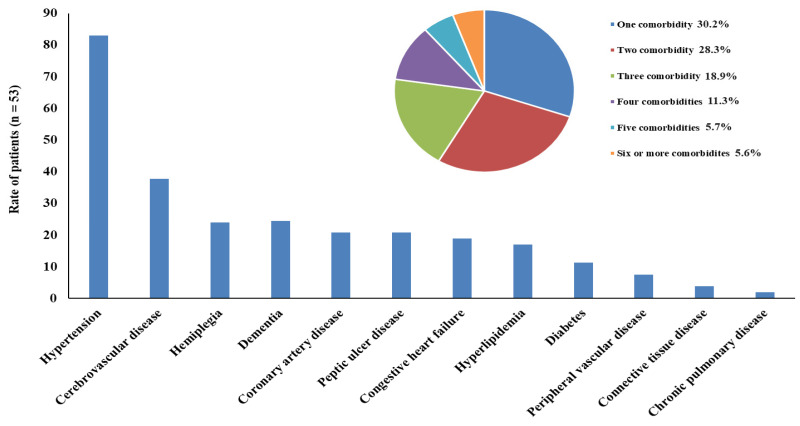
Distribution of comorbid conditions.

The study included a total of 159 participants, divided equally into three groups: individuals with epilepsy without comorbidities (*n* = 53), individuals with epilepsy and comorbidities (*n* = 53), and health controls (*n* = 53). Conditions such as hypertension, cerebrovascular disease, hemiplegia, dementia, coronary artery disease, peptic ulcer disease, congestive heart failure, hyperlipidemia, DM, peripheral vascular disease, connective tissue disorders, and chronic pulmonary diseases are classified as comorbidities. The distribution of comorbid conditions is presented in Figure 1.

The mean age was higher in the epilepsy group with comorbidities (56.2 ± 13.8 years) compared to the other groups, while it was lower in the epilepsy group without comorbidities (33 ± 12.5 years) than in the control group (44 ± 14.2 years) (*p* < 0.001). In terms of sex distribution, there were no significant differences between groups (*p* = 0.369). Regarding epilepsy classification, focal epilepsy was more common than generalized epilepsy across both patient groups, with a higher proportion in the epilepsy-with-comorbidities group (75.5%) compared to the epilepsy-without-comorbidities group (64.2%). Seizure frequency was higher in epilepsy patients with comorbidities compared to patients without comorbidities (75.5% vs. 54.7%, *p* < 0.001). Demographic and clinical findings of the study population are shown in detail in Table 1.

**Figure 2:**
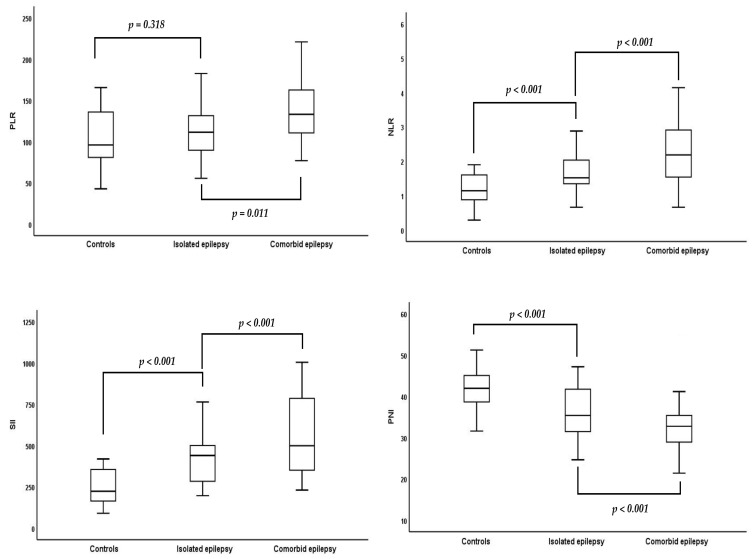
Comparison of platelet-to-lymphocyte ratio (PLR), neutrophil-to-lymphocyte ratio (NLR), systemic immune-inflammation index (SII), and prognostic nutritional index (PNI) among control, isolated epilepsy, and comorbid epilepsy groups.

Mean leukocyte count (*p* < 0.001), median neutrophil count (*p* < 0.001), mean platelet distribution width (PDW) (*p* = 0.005), and median C-reactive protein (CRP) (*p* = 0.045) levels were significantly higher in epilepsy patients with comorbidities. Similarly, inflammatory indices, such as the PLR (*p* < 0.001), NLR (*p* < 0.001), and SII (*p* < 0.001), were significantly elevated (Figure 2). The PNI was 36.5 ± 7.6 in the diagnosed with isolated epilepsy participants, 32.2 ± 6.7 comorbid diseases along with epilepsy participants, and 42.2 ± 4.7 in the control group (*p* < 0.000) (Figure 2) (Table 1).

**Figure 3:**
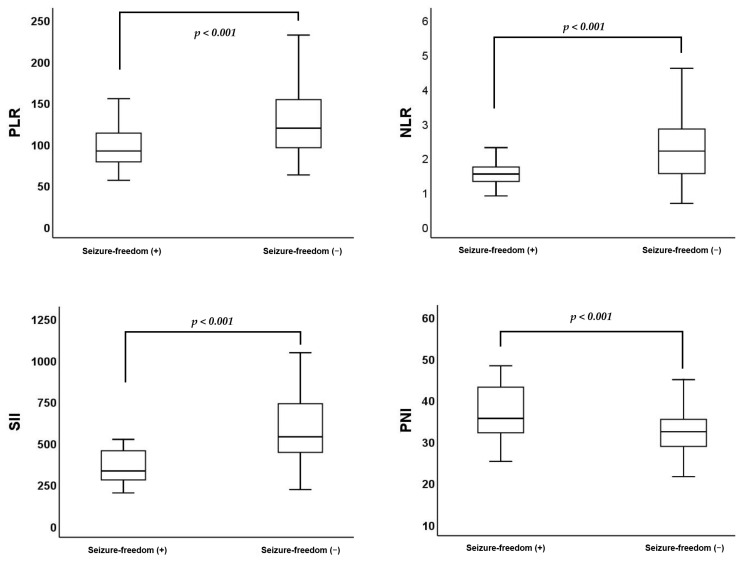
Distribution of platelet-to-lymphocyte ratio (PLR), neutrophil-to-lymphocyte ratio (NLR), systemic immune-inflammation index (SII), and prognostic nutritional index (PNI) according to seizure-freedom status.

The mean age and median mCCI were higher in patients with seizures compared to those without seizures. The proportion of patients with high comorbidity scores (≥5 points) was higher in patients with seizures compared to those without seizures. Median lymphocytes were lower; mean neutrophil, PLR, NLR, and SII levels were higher; and mean albumin and PNI levels were lower in patients with seizures compared to those without seizures (Figure 3) (Table 2).

Risk factors associated with seizure risk are shown in Table 3 in the univariable analysis. Accordingly, a high comorbidity score increased the probability of seizure risk by 4.56 times (OR = 4.56, *p* = 0.018). A one-unit increase in SII levels was found to increase the probability of seizure risk by 1.13 times (OR = 1.13, *p* < 0.001). A one-unit decrease in PNI levels was found to increase the probability of seizure risk by 1.14 (1/OR) times (OR = 0.88, *p* < 0.001).

**Figure 4:**
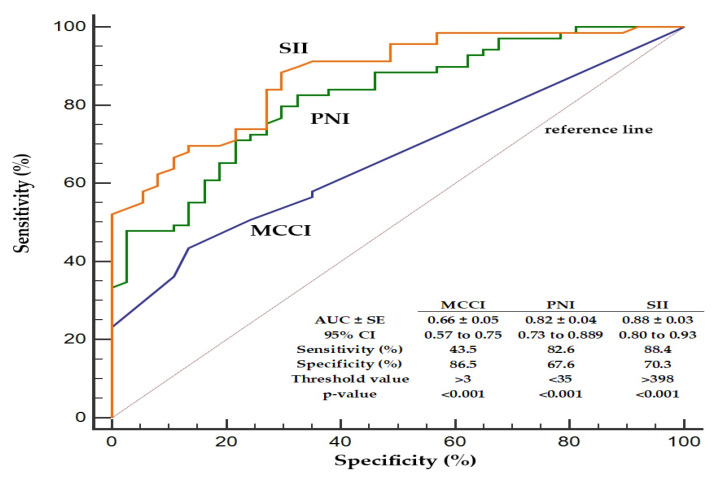
The diagnostic performance assessment of the systemic immune-inflammation index (SII) and prognostic nutritional index (PNI) with the Modified Charlson Comorbidity Index (MCCI) in predicting the seizure risk.

The ROC curve analysis demonstrated that the SII had the highest discriminative ability for seizure risk (AUC = 0.88), followed by the PNI (AUC = 0.82) and MCCI (AUC = 0.66). The SII and PNI showed high sensitivity, while the MCCI exhibited higher specificity. All three indices were statistically significant predictors (*p* < 0.001) (Figure 4).

The effect of comorbidity types on seizures in patients with comorbidities is shown in Table 4. Cerebrovascular accidents and hemiplegia were associated with the risk of seizures. When the effects of age, sex, epilepsy type, magnetic resonance imaging and electroencephalography findings, disease duration, and laboratory parameters were eliminated, the effect of cerebrovascular accident and hemiplegia on the risk of seizures remained significant. Accordingly, the probability of seizure risk was 4.15 times higher in those with cerebrovascular events compared to those without (OR = 4.15, *p* = 0.045). The probability of seizure risk was 4.48 times higher in those with hemiplegia compared to those without (OR = 4.48, *p* = 0.048).

**Figure 5:**
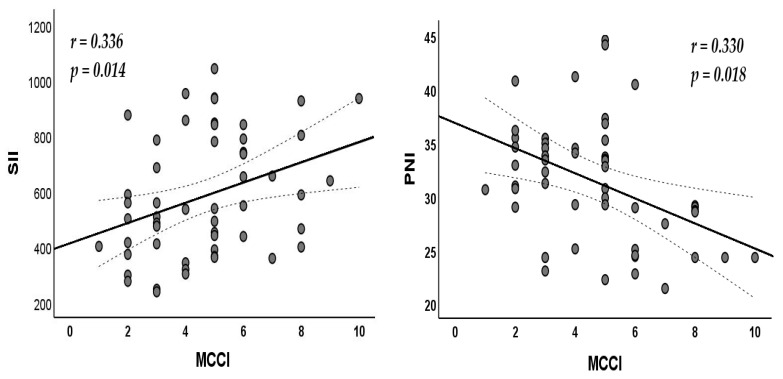
Correlation of systemic immune-inflammation index (SII) and prognostic nutritional index (PNI) with Modified Charlson Comorbidity Index (MCCI).

Factors associated with the mCCI in patients with comorbidities are shown in Table 5. Epilepsy type, magnetic resonance imaging findings, and electroencephalography findings were not associated with the mCCI. A positive correlation was observed between mCCI and SII levels, whereas PNI levels were negatively correlated with the mCCI (Figure 5).

## 4. Discussion

To our knowledge, this is the first study to evaluate the combined effects of hematological inflammatory markers and the mCCI on epilepsy prognosis. The results demonstrated that inflammatory parameters were significantly higher in epilepsy patients compared to healthy controls, with the highest levels observed in those with additional comorbidities. Furthermore, elevated mCCI scores, increased SII values, and the presence of cerebrovascular disease and hemiplegia were identified as key predictors of increased seizure risk. In addition, lower PNI values were consistently observed in epilepsy patients with comorbid conditions, suggesting that poor nutritional status may exacerbate the inflammatory response, and the associations suggest a role in worse clinical outcomes. This finding underscores the potential role of the PNI as a prognostic marker, highlighting its utility in identifying high-risk epilepsy patients and guiding individualized treatment strategies.

The mCCI, developed by Charlson et al. [27], is a scoring system that quantifies the severity of comorbid conditions, with higher scores reflecting greater comorbidity burden and mortality risk. It is widely used in clinical research to evaluate disease prognosis and survival outcomes. Similarly, hematological inflammatory parameters have emerged as potential prognostic markers in various diseases, including epilepsy. Previous studies have demonstrated that the MCCI is an effective tool for predicting prognosis across various disease populations. In line with these findings, our study identified a significant association between higher MCCI scores and increased seizure frequency in patients with epilepsy. The presence of comorbid conditions appears to exacerbate disease burden and complicate management in this population. Keezer et al. [29] evaluated the performance of three mortality risk-adjustment comorbidity indices in a community epilepsy cohort and found that the CCI was a valid predictor of mortality risk in epilepsy patients. These findings support our results, suggesting that a higher comorbidity burden, as measured by the MCCI, may suggest its role in worse clinical outcomes and reduced seizure control. Therefore, the MCCI may serve as a valuable clinical decision-making tool for identifying high-risk epilepsy patients and tailoring their management accordingly.

Several studies have explored the role of inflammatory markers in epilepsy. Hosseini et al. reported that NLR levels were significantly elevated in epilepsy patients during both the acute and subacute phases compared to healthy controls, with a notable difference between these phases [30]. In contrast, Özdemir et al. [31] found no significant difference in NLR and PLR levels between epilepsy patients and healthy individuals, which may be attributable to their small sample size of only 21 participants. Our findings, however, align with previous studies demonstrating that both NLR and PLR levels are elevated in epilepsy patients irrespective of comorbid conditions. In a study by Güneş et al. [32], a positive correlation was observed between epileptic seizures and both the NLR and PLR. Logistic regression analysis further indicated that NLR was a significant predictor of seizure development. Similarly, Shi et al. [33] reported that monocyte, lymphocyte, platelet, NLR, PLR, and monocyte-to-lymphocyte ratio (MLR) levels were elevated in patients with convulsive status epilepticus compared to healthy controls. Moreover, during active seizure periods, neutrophil, monocyte, lymphocyte, NLR, PLR, MLR, and SII levels were significantly higher than in post-treatment phases, with neutrophil counts declining in remission, which demonstrated that NLR is a reliable predictor of intensive care unit admission and prolonged hospital stay in status epilepticus patients, as confirmed by ROC analysis [34]. These findings collectively reinforce the notion that NLR serves as a prognostic marker for epilepsy severity and disease progression.

In a prospective cohort study involving 497,291 participants, Huang et al. [35] demonstrated that blood count-derived immunoinflammatory markers, including the NLR, PLR, and SII, were significantly associated with an increased risk of epilepsy. Consistent with these findings, our study also found elevated levels of the NLR, PLR, and SII in epilepsy patients with comorbidities, supporting the role of systemic inflammation in epileptogenesis. Consistent with previous studies, our results show that white blood cell count, neutrophils, NLR, PLR, and SII levels are significantly elevated in epilepsy patients compared to healthy controls. Discrepancies among studies regarding the significance of these inflammatory markers may stem from variations in sample size, patient disease status (e.g., prolonged remission periods), or the absence of comorbid conditions. Given that epileptic seizures involve complex processes, such as disruptions in extracellular ion homeostasis, altered energy metabolism, receptor dysfunction, and neurotransmitter imbalances, it is plausible that these events trigger chronic inflammatory pathways.

A key finding of our study is the positive correlation between the SII and mCCI, emphasizing the interplay between systemic inflammation and comorbid disease burden in epilepsy. Hypertension and cerebrovascular disease were the most frequently observed comorbidities, both of which are known to be associated with chronic inflammation, atherosclerosis, endothelial dysfunction, and target organ damage [36,37]. Previous research has consistently demonstrated that hematological inflammatory parameters are elevated in patients with hypertension, cerebrovascular disease, and other cardiovascular conditions compared to healthy controls [38]. Our results align with these findings, further suggesting that inflammation plays a crucial role in epilepsy pathophysiology, particularly in patients with comorbidities.

Beyond comorbid conditions, inflammatory mediators are released by glial cells, neurons, endothelial cells of the blood–brain barrier, and peripheral immune cells in response to epileptic activity. These inflammatory response associations suggest a role in both the onset and persistence of seizures across various epilepsy subtypes [39,40,41]. Notably, our regression analysis revealed that high mCCI and SII scores are significant risk factors for seizure development. Furthermore, when stratifying the study population by seizure status, we found that neutrophil, PLR, NLR, and SII levels were significantly lower, while lymphocyte levels were higher in the seizure-free group (patients without seizures for 12 months) compared to those who continued to experience seizures. These findings reinforce the hypothesis that chronic inflammation influences epilepsy progression and treatment outcomes.

The importance of considering how anti-epileptic drugs (AEDs) may influence systemic inflammation in epilepsy patients. Valproate (VPA), a commonly used AED, has been shown to modulate inflammatory pathways. Studies indicate that VPA can inhibit the activation of nuclear factor kappa B (NF-κB), a transcription factor involved in the expression of pro-inflammatory cytokines such as TNF-α and IL-6. By suppressing NF-κB activation, VPA reduces the production of these cytokines, thereby exerting anti-inflammatory effects [42]. Furthermore, research involving children with epilepsy demonstrated that treatment with valproate led to a significant decrease in serum levels of C–C motif ligand 2 (CCL2), a chemokine associated with inflammation, without altering levels of interleukin-1 beta (IL-1β) [43]. Guenther et al. [44] conducted a prospective study examining the effects of chronic VPA and levetiracetam (LEV) treatment on cytokine levels in humans. Their findings indicated that neither VPA nor LEV treatment significantly influenced the serum levels of interleukin-1 beta (IL-1β), interleukin-6 (IL-6), tumor necrosis factor-alpha (TNF-α), or monocyte chemoattractant protein-1 (MCP-1). However, VPA intake resulted in a significant decrease in the total white blood cell count and neutrophil percentage, while LEV treatment led to a decrease in the percentage of CD4+CD25+ T lymphocytes. These findings suggest that while VPA and LEV may modulate certain aspects of the immune system, they do not significantly alter systemic inflammatory cytokine levels. This contrasts with our study, where we observed elevated inflammatory hematological parameters, particularly the SII and MCCI scores, in epilepsy patients with comorbidities. The discrepancy between these studies may be attributed to differences in study design, patient populations, and the specific inflammatory markers assessed. Considering these differences, it is crucial to consider the potential impact of AEDs on inflammatory markers when interpreting the results of studies examining systemic inflammation in epilepsy. Further research is needed to elucidate the complex interactions between AEDs, the immune system, and inflammatory markers in epilepsy patients. While our study did not specifically address the impact of AEDs on inflammatory markers, including the NLR, we recognize the relevance of this aspect. Future research could benefit from examining the effects of AEDs, such as valproate, on the NLR and other inflammatory markers to better understand their role in modulating systemic inflammation in epilepsy patients.

The PNI has been increasingly recognized as an important marker for assessing both nutritional and immune status, influencing clinical outcomes in a variety of diseases. The PNI, which incorporates serum albumin levels and lymphocyte count, has been shown to correlate with survival rates and disease prognosis in patients with various conditions, including cancer, cardiovascular diseases, and chronic illnesses [45,46,47]. Inadequate nutritional intake is common in epilepsy patients and is associated with poorer seizure control and overall health status. This supports the notion that nutritional status plays a key role in epilepsy management [48,49]. Lower nutritional quality may suggest a role in increased seizure frequency, aligning with our study’s findings that low PNI values reflecting poor nutritional and immune status are more prevalent in patients with comorbid epilepsy. Children with refractory epilepsy are at a high risk of malnutrition, emphasizing the need for regular nutritional assessment in this population [50]. This finding supports our results, where low PNI scores in epilepsy patients with comorbidities suggest that compromised nutritional status may negatively impact disease prognosis. Therefore, our findings suggest that the PNI could serve as a useful marker to identify high-risk epilepsy patients, enabling more targeted and personalized treatment strategies aimed at improving both nutritional support and seizure management. Malnutrition, characterized by an inadequate intake of essential nutrients, leads to a decrease in serum albumin levels and lymphocyte counts. This nutritional deficiency impairs immune responses and increases systemic inflammation, as evidenced by elevated levels of inflammatory markers such as CRP and NLR [51]. Systemic inflammation can activate microglia and astrocytes in the central nervous system, leading to the release of pro-inflammatory cytokines and excitatory neurotransmitters. This neuroinflammatory environment enhances neuronal excitability and synaptic plasticity, thereby lowering the seizure threshold and increasing the risk of seizure occurrence [52]. Furthermore, malnutrition-induced alterations in mitochondrial function and oxidative stress can exacerbate neuronal vulnerability [29]. These factors’ associations suggest a role in neuronal hyperexcitability and increase susceptibility to seizures. In summary, a low PNI reflects a state of malnutrition and systemic inflammation, both of which can adversely affect neuronal function and increase seizure risk. Monitoring the PNI in epilepsy patients may provide valuable insights into their nutritional and immune status, aiding in the identification of individuals at higher risk for seizures and guiding appropriate therapeutic interventions.

Several limitations should be acknowledged in interpreting the results of this study. The retrospective nature of the data collection may limit the ability of establishing causality between inflammatory markers, comorbidity burden, and epilepsy prognosis. Moreover, the cross-sectional evaluation of hematological parameters precludes the analysis of longitudinal changes or fluctuations in inflammation over time. The relatively small and single-center sample may not fully represent the broader epilepsy population, particularly regarding diverse etiologies and treatment backgrounds. Potential confounders, such as anti-epileptic drug use, nutrition, and underlying infections, were not fully controlled, which may have affected the inflammatory markers measured. Another important limitation of this study is that age and sex matching between the groups, although necessary to reduce confounding, restricted our ability to assess the independent effects of these two variables on seizure frequency, inflammatory markers, and nutritional status. This study did not assess unmeasured confounders, such as socioeconomic status and adherence to antiepileptic drugs (AEDs), which can influence both inflammatory markers and epilepsy outcomes.

## 5. Conclusions

In conclusion, this study demonstrated that hematological inflammatory parameters were elevated in patients with epilepsy compared to healthy controls, with further increases observed in those with additional comorbidities. Both the presence of comorbid conditions and elevated SII levels were identified as significant risk factors for seizure development. The PNI, a practical and cost-effective tool for evaluating nutritional and immune status, was also assessed in this context. Our findings revealed that epilepsy patients, particularly those with comorbidities, exhibited lower PNI values, suggesting an increased risk of malnutrition. These results highlight the potential utility of the PNI as a supplementary marker in the clinical monitoring and management of epilepsy. Future studies should focus on validating PNI and SII cutoffs in multicenter charts to enhance their clinical applicability in epilepsy management. Additionally, prospective trials should evaluate the efficacy of anti-inflammatory therapies in patients with high MCCI scores to determine their potential in improving seizure control and overall patient outcomes.

## Figures and Tables

**Table 1:** Demographic and clinical findings.

Variables	Control *n* = 53	Epilepsy		*p*-Value
		Without Comorbidity *n* = 53	With Comorbidity *n* = 53	
Age, years	**44.0 ± 14.2**	**33.0 ± 12.5**	**56.2 ± 13.8**	<0.001 *
Sex, *n* (%)				
Female	21 (39.6)	28 (52.8)	22 (41.5)	0.369
Male	32 (60.4)	25 (47.2)	31 (58.5)	
Epilepsy type, *n* (%)				
Focal	-	34 (64.2)	40 (75.5)	0.441
Generalized	-	14 (26.4)	9 (17.0)	
Combined	-	5 (9.4)	4 (17.5)	
MRI findings, *n* (%)				
Normal	-	41 (77.4)	31 (58.5)	0.060
Abnormal	-	12 (22.6)	22 (41.5)	
EEG, *n* (%)				
Normal	-	4 (7.5)	5 (9.4)	0.999
Epileptiform	-	45 (84.9)	44 (83.0)	
Nonepileptiform	-	4 (7.5)	4 (7.5)	
Duration of disease, years	-	3 (2–9)	4 (2–7)	
mCCI	-	-	5 (3–6)	-
Low comorbidity	-	-	9 (17.0)	-
Moderate comorbidity	-	-	15 (28.3)	
High comorbidity	-	-	29 (54.7)	
Laboratory findings				
Hemoglobin, g/dL	**14.1 ± 1.5**	13.3 ± 1.3	13.6 ± 1.5	0.007 *
RBC, ×10^6^ µL	4.8 ± 0.6	**4.5 ± 0.4**	4.7 ± 0.7	0.031 *
Hematocrit, %	**42.0 ± 3.5**	39.2 ± 3.4	40.8 ± 3.5	<0.001 *
MCV, fL	86.6 ± 3.3	85.9 ± 5.0	85.3 ± 5.7	0.352
MCH, pg	29.8 ± 1.5	29.3 ± 1.9	**28.7 ± 2.0**	0.008 *
MCHC, g/dL	34.2 ± 1.6	33.8 ± 1.2	33.7 ± 1.3	0.183
Leukocytes, ×10^3^ µL	6.0 ± 1.5	6.8 ± 1.8	**8.0 ± 2.0**	<0.001 *
Lymphocytes, ×10^3^ µL	2.5 (1.9–2.7)	2.3 (1.8–2.6)	**2.1 (1.7–2.5)**	0.030 *
Neutrophils, ×10^3^ µL	**2.8 (2.3–3.1)**	**3.5 (3.0–4.6)**	**4.7 (4–5.8)**	<0.001 *
Monocytes, ×10^3^ µL	0.5 ± 0.1	0.6 ± 0.2	0.6 ± 0.2	0.222
Platelets, ×10^3^ µL	241.5 ± 70	262.6 ± 76.7	249.7 ± 65.9	0.306
MPV, fL	9.4 ± 1.1	9.6 ± 0.9	9.4 ± 1.2	0.424
PCT, %	0.2 ± 0.1	0.3 ± 0.1	0.2 ± 0.1	0.265
PDW, %	10.2 ± 1.8	10.4 ± 1.9	**11.7 ± 2.4**	0.005 *
CRP, mg/dL	0.5 (0.1–1.0)	0.5 (0.1–1.0)	**0.9 (0.5–2.3)**	0.045 *
Albumin, g/dL	**4.2 ± 0.5**	**3.7 ± 0.7**	**3.2 ± 0.6**	<0.001 *
PLR	95.9 (80.9–135.8)	111.4 (89.6–131.4)	**133.7 (114.2–168.1)**	0.044 *
NLR	**1.1 (0.9–1.6)**	**1.5 (1.3–2.0)**	**2.2 (1.5–2.9)**	<0.001 *
SII	**244.3 (185.0–376.3)**	**449.4 (283.4–504.1)**	**500 (371.5–787.7)**	<0.001 *
PNI	**42.2 ± 4.7**	**36.5 ± 7.6**	**32.2 ± 6.7**	<0.001 *
≥35 (normal)	35 (66.0)	23 (43.4)	16 (30.2)	<0.001 *
<35 (malnutrition)	18 (34.0)	30 (56.6)	37 (69.8)	
Seizure frequency, *n* (%)				
Seizure-freedom	-	24 (45.3)	13 (24.5)	0.020 *
Once a month	-	5 (9.4)	9 (17.0)	
More than once a month	-	5 (9.4)	8 (15.1)	
Low comorbidity	-	13 (24.5)	7 (13.2)	
Once a year	-	6 (11.3)	16 (30.2)	
More than once a year				

Data are mean ± standard deviation or median (IQR) or number (%). * *p* < 0.05 indicates statistical significance. Bold characters show differences between groups. Abbreviations: CRP, C-reactive protein; EEG, electroencephalography; mCCI, Modified Charlson Comorbidity Index; MCH, mean corpuscular hemoglobin; MCHC, mean corpuscular hemoglobin concentration; MCV, mean corpuscular volume; MPV, mean platelet volume; NLR, neutrophil-to-lymphocyte ratio; PCT, plateletcrit; PDW, platelet distribution width; PLR, platelet-to-lymphocyte ratio; PNI, prognostic nutritional index; RBC, red blood cell count; SII, systemic immune-inflammation index.

**Table 2:** Distribution of demographic and clinical findings in patients with and without seizures.

Variables	Seizure Freedom		*p*-Value
	Yes *n* = 37	No *n* = 69	
Age, years	38.8 ± 15.9	48.6 ± 16.8	0.004 *
Sex, *n* (%)			
Female	16 (43.2)	34 (49.3)	0.684
Male	21 (56.8)	35 (50.7)	
Epilepsy type, *n* (%)			
Focal	23 (62.2)	51 (73.9)	0.296
Generalized	9 (24.3)	14 (20.3)	
Combined	5 (13.5)	4 (5.8)	
MRI findings, *n* (%)			
Normal	28 (75.7)	44 (63.8)	0.276
Abnormal	9 (24.3)	25 (36.2)	
EEG, *n* (%)			
Normal	5 (13.5)	4 (5.8)	0.426
Epileptiform	29 (78.4)	60 (87.0)	
Nonepileptiform	3 (8.1)	5 (7.2)	
Duration of disease, years	3 (2–9)	4 (2–8)	0.883
mCCI	3 (2–5)	5 (3.5–6)	0.008 *
No comorbid conditions	24 (64.9)	29 (42.0)	0.022 *
Low comorbidity	4 (10.8)	5 (7.2)	
Moderate comorbidity	5 (13.5)	10 (14.5)	
High comorbidity	4 (10.8)	25 (36.2)	
Laboratory findings			
Hemoglobin, g/dL	13.1 ± 1.0	13.6 ± 1.6	0.053
RBC, ×10^6^ µL	4.6 ± 0.6	4.7 ± 0.5	0.290
Hematocrit, %	39.4 ± 3.4	40.2 ± 3.5	0.114
MCV, fL	85.4 ± 5.4	85.7 ± 5.4	0.788
MCH, pg	28.7 ± 1.8	29.2 ± 2.0	0.228
MCHC, g/dL	33.7 ± 1.3	33.8 ± 1.2	0.716
Leukocytes, ×10^3^ µL	7.1 ± 2.0	7.5 ± 1.9	0.291
Lymphocytes, ×10^3^ µL	2.6 (2.2–2.8)	2.1 (1.8–2.7)	0.014 *
Neutrophils, ×10^3^ µL	3.6 (3–4.7)	4.6 (3.5–5.7)	0.005 *
Monocytes, ×10^3^ µL	0.6 ± 0.2	0.6 ± 0.2	0.363
Platelets, ×10^3^ µL	239.3 ± 74.8	265.2 ± 68.4	0.075
MPV, fL	9.5 ± 1.1	9.5 ± 1.0	0.926
PCT, %	0.2 ± 0.1	0.2 ± 0.1	0.223
PDW, %	10.7 ± 3.0	11.3 ± 2.7	0.336
CRP, mg/dL	0.1 (0.1–1.2)	0.2 (0.1–1.5)	0.620
Albumin, g/dL	3.7 ± 0.7	3.2 ± 0.6	<0.001 *
PLR	91.0 (77.9–112.6)	118.6 (95.0–153.1)	<0.001 *
NLR	1.5 (1.3–1.7)	2.2 (1.5–2.8)	<0.001 *
SII	328.6 (274.4–449.4)	534 (439.6–733.8)	<0.001 *
PNI	37.6 ± 7.6	32.0 ± 6.5	<0.001 *
35≥ (normal)	21 (56.8)	18 (26.1)	<0.001 *
<35 (malnutrition)	16 (43.2)	51 (73.9)	

Data are mean ± standard deviation or median (IQR), or number (%). * *p* < 0.05 indicates statistical significance. Abbreviations: CRP, C-reactive protein; EEG, electroencephalography; mCCI, Modified Charlson Comorbidity Index; MCH, mean corpuscular hemoglobin; MCHC, mean corpuscular hemoglobin concentration; MCV, mean corpuscular volume; MPV, mean platelet volume; NLR, neutrophil-to-lymphocyte ratio; PCT, plateletcrit; PDW, platelet distribution width; PLR, platelet-to-lymphocyte ratio; PNI, prognostic nutritional index; RBC, red blood cell count; SII, systemic immune-inflammation index.

**Table 3:** Independent predictors of seizure risk.

Variables	Univariable Regression		Multivariable Regression	
	OR (95% CI)	*p*-Value	OR (95% CI)	*p*-Value
Age, years	1.04 (1.01–1.07)	0.006 *	-	-
mCCI	1.68 (1.10–2.56)	0.016 *	-	-
No comorbid conditions	ref		ref	
Low comorbidity	1.03 (0.25–4.28)	0.963	0.46 (0.09–2.33)	0.650
Moderate comorbidity	1.66 (0.50–5.51)	0.411	1.52 (0.41–5.65)	0.528
High comorbidity	5.17 (1.58–16.93)	0.007 *	4.56 (1.30–16.01)	0.018 *
Laboratory findings				
Lymphocytes	0.48 (0.26–0.85)	0.013 *	-	-
Neutrophils	1.47 (1.10–1.98)	0.010 *	-	-
Albumin	0.32 (0.17–0.61)	0.001 *	-	-
PLR	1.03 (1.02–1.05)	<0.001 *	-	-
NLR	4.35 (1.96–9.63)	<0.001 *	-	-
SII	1.13 (1.08–1.19)	<0.001 *	1.13 (1.08–1.19)	<0.001 *
PNI	0.89 (0.84–0.95)	<0.001 *	0.88 (0.81–0.96)	0.004 *
			Nagelkerke R^2^ = 0.59	

* *p* < 0.05 indicates statistical significance. Abbreviations: CI, confidence interval; mCCI, Modified Charlson Comorbidity Index; NLR, neutrophil-to-lymphocyte ratio; PLR, platelet-to-lymphocyte ratio; SII, systemic immune-inflammation index; OR, odds ratio.

**Table 4:** Comorbidities associated with seizure risk in patients with comorbidity.

Variables	Crude Regression		Adjusted Regression	
	OR (95% CI)	*p*-Value	OR (95% CI)	*p*-Value
Hypertension	1.70 (0.36–8.05)	0.504	1.92 (0.38–9.76)	0.432
Cerebrovascular disease	4.5 (1.08–22.97)	0.021 *	4.15 (1.10–20.30)	0.045 *
Hemiplegia	5.14 (1.06–24.12)	0.025 *	4.48 (1.05–21.6)	0.048 *
Dementia	0.40 (0.10–1.55)	0.187	0.25 (0.04–1.07)	0.106
Coronary artery disease	2.08 (0.39–10.59)	0.385	1.03 (0.09–12.17)	0.981
Peptic ulcer disease	0.48 (0.11–2.00)	0.312	0.42 (0.09–1.95)	0.271
Congestive heart failure	1.11 (0.25–4.86)	0.889	1.17 (0.25–5.44)	0.841
Hyperlipidemia	3.48 (0.40–30.54)	0.26	3.22 (0.36–29.14)	0.299
Diabetes mellitus	1.71 (0.18–16.18)	0.638	1.22 (0.11–13.45)	0.872
Peripheral vascular disease	1.33 (0.14–13.12)	0.805	1.36 (0.13–14.80)	0.799
Connective tissue disease	0.63 (0.05–7.59)	0.717	0.98 (0.07–13.75)	0.989
Chronic pulmonary disease	0.31 (0.02–5.30)	0.417	0.19 (0.01–3.72)	0.275

* *p* < 0.05 indicates statistical significance. Abbreviations: CI, confidence interval; OR, odds ratio.

**Table 5:** Findings associated with the Modified Charlson Comorbidity Index (MCCI).

Variables	mCCI	
	r	*p*
Age	0.479	<0.001 *
Sex		
Female	5 (3–6)	0.355
Male	5 (3–6)	
Epilepsy type		
Focal	5 (3–6)	0.806
Generalized	5 (4–6)	
Combined	5 (4–6)	
MRI findings		
Normal	4 (3–5)	0.280
Abnormal	5 (3–6)	
EEG		
Normal	4 (3–5)	0.648
Epileptiform	5 (3–6)	
Nonepileptiform	4 (3–5)	
Duration of disease	−0.058	0.683
Laboratory findings		
Hemoglobin	0.098	0.484
RBC	0.168	0.230
Hematocrit	−0.059	0.673
MCV	0.126	0.370
MCH	0.192	0.168
MCHC	−0.039	0.784
Leukocytes	0.169	0.225
Lymphocytes	−0.069	0.622
Neutrophils	0.066	0.637
Monocytes	0.154	0.271
Platelets	0.189	0.174
MPV	0.092	0.513
PCT	0.025	0.858
PDW	0.068	0.633
CRP	0.075	0.594
Albumin	−0.292	0.089
PLR	0.226	0.104
NLR	0.126	0.369
SII	0.336	0.014 *
PNI	−0.330	0.018 *

* *p* < 0.05 indicates statistical significance. Abbreviations: CRP, C-reactive protein; EEG, electroencephalography; mCCI, Modified Charlson Comorbidity Index; MCH, mean corpuscular hemoglobin; MCHC, mean corpuscular hemoglobin concentration; MCV, mean corpuscular volume; MPV, mean platelet volume; NLR, neutrophil-to-lymphocyte ratio; PCT, plateletcrit; PDW, platelet distribution width; PLR, platelet-to-lymphocyte ratio; RBC, red blood cell count; SII, systemic immune-inflammation index.

## Data Availability

The data underlying this article are available in the article. If needed, please contact the corresponding author. The email address is demetaygun@yahoo.com.
